# Pt-free carbon-based fuel cell catalyst prepared from spherical polyimide for enhanced oxygen diffusion

**DOI:** 10.1038/srep23276

**Published:** 2016-03-18

**Authors:** Yuta Nabae, Shinsuke Nagata, Teruaki Hayakawa, Hideharu Niwa, Yoshihisa Harada, Masaharu Oshima, Ayano Isoda, Atsushi Matsunaga, Kazuhisa Tanaka, Tsutomu Aoki

**Affiliations:** 1 Department of Organic and Polymeric Materials, Tokyo Institute of Technology, 2-12-1 S8-26 Ookayama, Meguro-ku, Tokyo 152-8552, Japan; 2 Institute for Solid State Physics, The University of Tokyo, 5-1-5 Kashiwanoha, Kashiwa, Chiba 277-8581, Japan; 3 Synchrotron Radiation Research Organization, The University of Tokyo, SPring-8, 1-1-1, Koto, Sayo-cho, Sayo-gun, Hyogo 679-5198, Japan; 4 Toshiba Fuel Cell Power Systems Corporation, 4-1 Ukishima-cho, Kawasaki-ku, Kawasaki-shi, Kanagawa 210-0862 Japan

## Abstract

The development of a non-precious metal (NPM) fuel cell catalyst is extremely important to achieve globalization of polymer electrolyte fuel cells due to the cost and scarcity of platinum. Here, we report on a NPM cathode catalyst prepared by the pyrolysis of spherical polyimide nanoparticles that contain small amounts of Fe additive. 60 nm diameter Fe-containing polyimide nanoparticles were successfully synthesized by the precipitation polymerization of pyromellitic acid dianhydride and 1,3,5-tris(4-aminophenyl)benzene with Fe(acac)_3_ (acac = acetylacetonate) as an additive. The particles were subsequently carbonized by multistep pyrolysis to obtain the NPM catalyst while retaining the small particle size. The catalyst has good performance and promising durability for fuel cell applications. The fuel cell performance under a 0.2 MPa air atmosphere at 80 °C of 1.0 A cm^−2^ at 0.46 V is especially remarkable and better than that previously reported.

Polymer electrolyte fuel cells (PEFCs) have received a great deal of attention due to their high energy conversion efficiency and have been commercialized in automobile and combined heat and power (CHP) applications. In commercialized PEFCs, both the anode and cathode, which oxidize hydrogen and reduce oxygen, respectively, utilize platinum-based catalysts to ensure a sufficiently high reaction rate. However, the cost and scarcity of platinum is a major obstacle to the globalization of PEFCs; therefore, it is necessary to develop non-precious metal (NPM) cathode catalysts. Since Jasinski discovered the catalytic activity of cobalt phthalocyanine^1^ for the oxygen reduction reaction (ORR) and Jahnke *et al.* reported the heat treatment of cobalt dibenzotetraazaannulene (CoTAA)^2^, numerous attempts have been made to develop NPM cathode catalysts by the pyrolysis of precursors that contain transition metals (mainly Fe or Co), a nitrogen source, and a carbon source^3^,^4^,^5^,^6^,^7^,^8^,^9^,^10^. Promising fuel cell performance has been reported for several NPM catalysts; however, the majority of these reports involved the use of pure oxygen for the cathode gas. Therefore, considering that air would be utilized for the cathode gas in most practical PEFC applications, it is very important to design the cathode layer to minimize the polarization caused by the transport of oxygen molecules.

In this context, our research group is interested in the morphological control of NPM catalysts to maximize the fuel cell performance under air. Here, we report on a NPM cathode catalyst prepared by the pyrolysis of spherical polyimide nanoparticles that contain small amounts of Fe additive. We have already reported that many nitrogen containing polymers, such as polyimides and polyamides, can be converted into catalytically active carbon materials by pyrolysis at high temperatures^11^. In addition, the morphology of polyimide can be controlled through precipitation polymerization, and the fine particle morphology can be retained after carbonization^12^. Here, we report on the synthesis of fine polyimide nanoparticles with diameters around 60 nm, and the catalytic performance of the carbonized particles as a cathode material under fuel cell conditions using air.

## Results and Discussion

**Figure 1:**
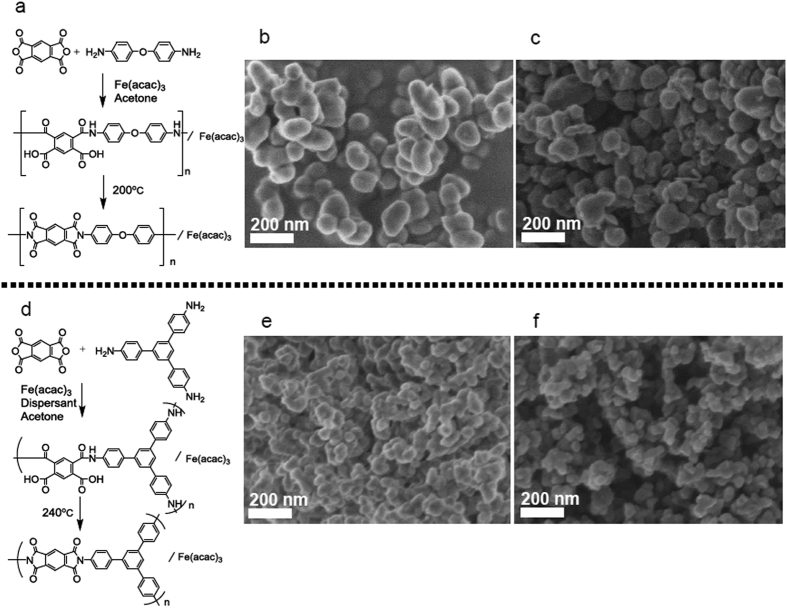
(**a**) Synthetic route for the polyimide and FE-SEM images of the polyimide particles (**b**) before and (**c**) after carbonization to produce Fe/PI(100)-1000-III-NH_3_. (**d**) Synthetic route for the polyimide and FE-SEM images of the polyimide particles (**e**) before and (**f**) after carbonization to produce Fe/PI(60)-1000-III-NH_3_.

Figure 1 shows the synthesis route for the preparation of the polyimide nanoparticles and FE-SEM images of the as-prepared and carbonized nanoparticles. Synthesis from pyromellitic acid dianhydride (PMDA) and 4,4′-oxydianiline (ODA), which are the most common polyimide precursors (Fig. 1a), resulted in polyimide nanoparticles with diameters of approximately 100 nm (Fig. 1b). The obtained Fe-containing polyimide nanoparticles were carbonized by multistep pyrolysis, where the Fe species catalyzes carbonization up to 600 °C and excess Fe species are removed before treatment at even higher temperatures to minimize the loss of nitrogen species at higher temperature^13^,^14^. The diameters of the carbonized samples were similar to those of the polyimide particles (Fig. 1c). To obtain even smaller polyimide particles, the precursor and polymerization conditions were optimized, whereby polyimide nanoparticles with diameters of 60 nm were synthesized by employing a triamine monomer, 1,3,5-tris(4-aminophenyl)benzene (TAPB), instead of ODA in the presence of a dispersant (Fig. 1d,e). These polyimide nanoparticles were also carbonized using a multistep pyrolysis process and the diameter was successfully retained (Fig. 1f). The carbonized particles obtained from the 100 nm and 60 nm diameter polyimide nanoparticles are denoted as Fe/PI(100)-1000-III-NH_3_ and Fe/PI(60)-1000-III-NH_3_, respectively.

**Figure 2:**
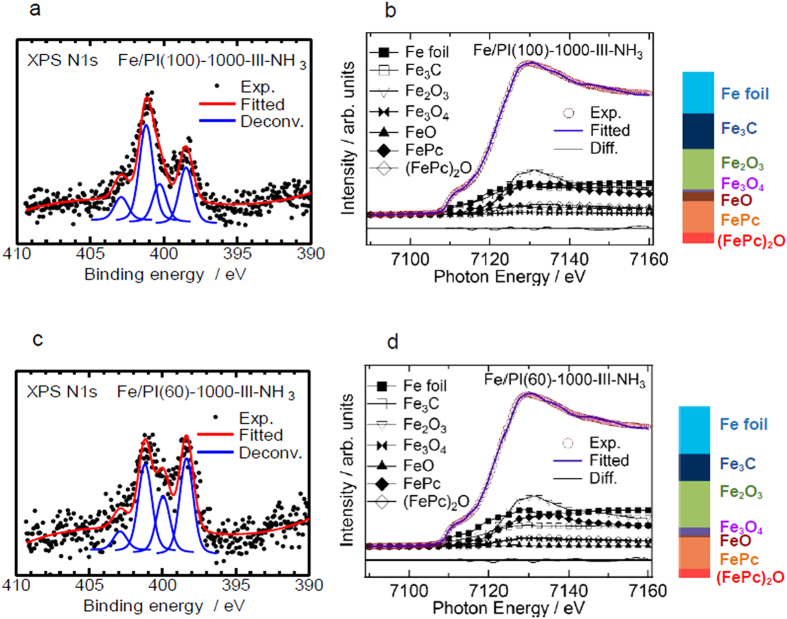
(**a**) N 1s XPS spectrum with deconvoluted curves and (**b**) Fe K-edge XANES spectrum with reference spectra weighted by their contribution to Fe/PI(100)-1000-III-NH_3_. (**c**) N 1s XPS and (**d**) Fe K-edge XANES spectra for Fe/PI(60)-1000-III-NH_3_. The bar graphs on the right provide visual representations of the relative compositions.

The prepared catalyst samples were characterized using several spectroscopic and analytical methods, the results of which are summarized in Table 1 and Fig. 2. According to the N_2_ adsorption measurements, the isotherms of which are shown in Figure S1, these samples show similar specific surface areas but Fe/PI(60)-1000-III-NH_3_ shows a slightly higher total pore volume, probably reflecting the smaller particle size. The nitrogen contents were determined to be 2–3 wt% by CHN elemental analysis (Table 1). A more detailed analysis for the nitrogen species was carried out using X-ray photoelectron spectroscopy (XPS), the results of which are shown in Fig. 2a,c. The N 1*s* spectra were deconvoluted into four peaks: pyridinic (398.4–398.5 eV), pyrrolic (400.0–400.3 eV), graphitic (401.2 eV) and oxidized (402.9 eV) nitrogen species^15^. The two largest peaks were assigned to pyridinic and graphitic nitrogen species in both the Fe/PI(100)-1000-III-NH_3_ and Fe/PI(60)-1000-III-NH_3_ catalysts. These nitrogen species have been proposed as catalytically active sites for the ORR^16^,^17^, although they may be active for 2-electron reduction rather than 4-electron reduction in acidic media^18^. The peak at 398.4–398.5 eV may obscure the signal from any FeN_*x*_ type structures, if present, which has been reported to be at 398.7 eV^19^. The Fe contents were determined by electron probe micro analysis (EPMA) to be 1.1–1.5 wt%. A more detailed analysis for the Fe species was conducted using powder X-ray diffraction and X-ray adsorption near edge structure (XANES) spectroscopy. The XRD patterns (Figure S2) suggest that both samples contain considerable amount of iron carbide. Linear combination fitting analyses of the XANES spectra are shown in Fig. 2b,d. The majority of Fe species can be assigned to clustered Fe species such as metallic iron, carbide and oxide. In addition, certain amounts of mononuclear Fe species, such as FePc-like (Fe with *D*_4*h*_ symmetry) and (FePc)_2_O-like (Fe with *C*_4*v*_ symmetry), are probably formed. The ratio of these FeN_*x*_ species (Fe with *D*_4*h*_ or *C*_4*v*_ symmetry) are almost the same between Fe/PI(100)-1000-III-NH_3_ and Fe/PI(60)-1000-III-NH_3_. These XPS and XANES data suggest the co-existence of Fe-free nitrogen species (pyridinic and graphitic), and FeN_*x*_ species in both Fe/PI(100)-1000-III-NH_3_ and Fe/PI(60)-1000-III-NH_3_. It should be noted that these two catalysts have similar surface properties and surface areas but different particle sizes.

**Figure 3:**
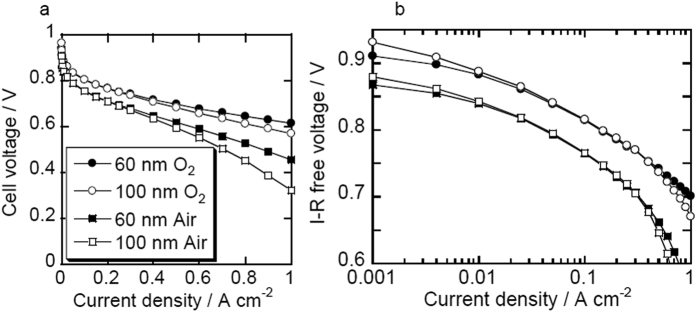
(**a**) *I*-*V* performance curves under 0.2 MPa air atmosphere and (**b**) Tafel plots of the *I*-*V* curves with the Fe/PI(100)-1000-III-NH_3_ and Fe/PI(60)-1000-III-NH_3_ cathode catalysts. Anode: PtRu/C catalyst with 0.4 mg-PtRu cm^−2^ loading, humidified H_2_ at 80 °C. Cathode: 4 mg cm^−2^ catalyst loading, pure or balanced O_2_ (humidified) at 80 °C. Electrolyte: Nafion NR211. *T*: = 80 °C.

**Figure 4:**
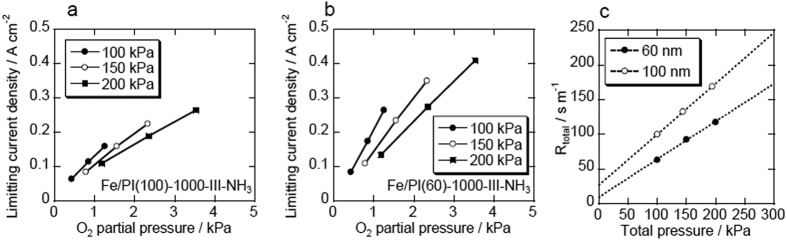
(**a,b**) The effect of O_2_ partial pressure on the limiting current density and (**c**) total gas transport resistance with the Fe/PI(100)-1000-III-NH_3_ and Fe/PI(60)-1000-III-NH_3_ cathode catalysts. Anode: PtRu/C catalyst with 0.4 mg-PtRu cm^−2^ loading, humidified H_2_ at 80 °C. Cathode: 4 mg cm^−2^ catalyst loading, pure or balanced O_2_ (humidified) at 80 °C. Electrolyte: Nafion NR211. *T*: = 80 °C.

The catalysts were tested under practical fuel cell conditions. Figure 3a shows *I*-*V* performance curves for the membrane electrode assembly (MEA) prepared using Fe/PI(100)-1000-III-NH_3_ and Fe/PI(60)-1000-III-NH_3_ as cathode catalysts. The MEA with the Fe/PI(100)-1000-III-NH_3_ cathode showed open circuit voltages of 0.96 and 0.90 V under pure O_2_ and air, respectively, and the current density reached 1 A cm^−2^ at 0.57 V (O_2_) and 0.32 V (air). The MEA with the Fe/PI(60)-1000-III-NH_3_ cathode showed similar open circuit voltages of 0.94 V (O_2_) and 0.90 V (air), but higher voltages of 0.62 V (O_2_) and 0.46 V (air) at a current density of 1 A cm^−2^. The performance of the MEA with Fe/PI(60)-1000-III-NH_3_ under pure O_2_ is similar to that of the state-of-the-art NPM cathodes^5^. To the best of our knowledge, the performance of the Fe/PI(60)-1000-III-NH_3_ catalyst cathode under an air atmosphere is better than any other reported. Although, there have been few *I*-*V* performance curves demonstrated with NPM cathode catalysts under air, that for state-of-the-art NPM cathodes in air is approximately 1.0 A cm^−2^ at 0.38 V under a total pressure of 0.25 MPa, as reported by Mukerjee and coworkers^9^. Fe/PI(60)-1000-III-NH_3_ demonstrates a current density of 0.8 A cm^−2^ at 0.5 V under a lower total pressure of 0.2 MPa. Figure 3b shows Tafel plots for the MEAs with both of the prepared catalysts. The plots suggest that the main difference between the performances for these two catalysts is derived from mass transport diffusion at high current density rather than the kinetics at low current density. To highlight the improvement in mass transport, the effect of gas pressure on the limiting current was analyzed using the method proposed by Shinohara and coworkers^20^,^21^. Under the condition in which O_2_ transport limits the current density, the limiting current *i*_lim_, is proportional to the partial pressure of O_2_:


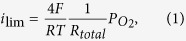


where *R*_total_ (s m^−1^) is the total gas transport resistance and *P*_O2_ is the partial pressure of O_2_. *R*_total_ can be described as:


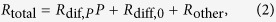


where *R*_dif,p_ is the molecular diffusion resistance, which is a function of the total gas pressure, *R*_dif,0_ is the molecular diffusion resistance when the total gas pressure is assumed to be zero, and *R*_other_ is the transport resistance through other transport processes. To analyze the effect of the particle size on the O_2_ transport, *i*_lim_ was measured at a cell voltage of 0.2 V and plotted against *P*_O2_, and the slope was then converted into *R*_total_ (Fig. 4a,b). The measurements were performed under several total gas pressures and the results are shown in Fig. 4c. The slope of the plot for the smaller particle size (60 nm) cathode catalyst was significantly decreased from 0.73 to 0.54 s m^−1^ kPa^−1^, which suggests a significant improvement of O_2_ diffusion in the NPM catalyst layer.

**Figure 5:**
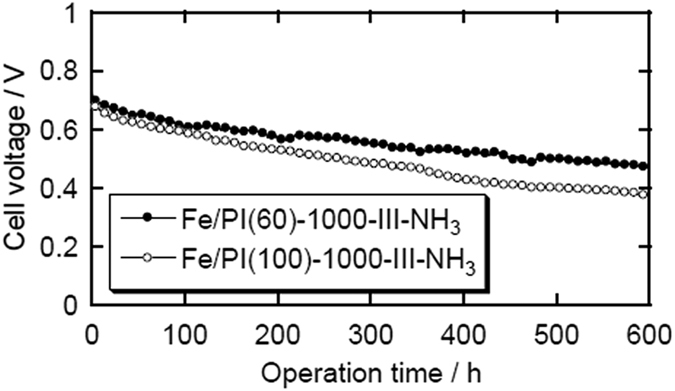
Cell voltage stability curves at 0.2 A cm^−2^ with air as the cathode gas. The conditions were the same as those detailed in Fig. 3.

The durability of the MEAs was investigated by operating the cells for a long time. Figure 5 shows the changes in the cell voltage during operation at 0.2 A cm^−2^ for 600 h. Although the cell voltage certainly decreased, the cells were successfully operated for 600 h. To our knowledge, these are some of the longest durability tests performed using NPM cathode catalysts, whereas most of the state-of-the-art NPM catalysts are significantly degraded within 100 h under practical fuel cell conditions. Thus, the durability demonstrated for the NPM catalyst cathodes in this study is quite promising.

The reaction mechanism and active sites of this type of catalyst are still under debate. One conventional explanation is that the ORR over the Fe-based catalysts proceeds via direct 4-electron reduction (Figure S3) on FeN_x_ centers^3^. In contrast, Attanasov and colleagues have proposed 2 + 2 electron reduction pathways, each of which may be catalyzed by different active sites^22^. Quite recently, Ohsaka and colleagues have also proposed such a series reduction pathway through investigation of an NPM catalyst prepared in the same manner as that for Fe/PI(100)-1000-III-NH_3_ in this study^23^,^24^,^25^. Several experimental evidences have suggested that the first two electron reduction to form H_2_O_2_ is catalyzed by Fe-free nitrogen doped carbon^18^,^26^; however, the second two electron reduction might be catalyzed by Fe containing active sites. Indeed, Ohsaka and colleagues confirmed that a Fe-free carbon-based catalyst prepared from polyimide catalyzes the first 2-electron reduction to form H_2_O_2_, while the consumption of H_2_O_2_ and the onset potential increase by the post-formation of a FeN_x_ species, the structure of which was confirmed by synchrotron radiation spectroscopy^27^. In this study, we have confirmed the co-existence of Fe-free nitrogen species (pyridinic and graphitic), and FeN_*x*_ species in both of Fe/PI(100)-1000-III-NH_3_ and Fe/PI(60)-1000-III-NH_3_ catalysts, which suggests that these species could catalyze one or more steps in a 2 + 2 electron reduction process.

In summary, a highly active NPM cathode catalyst, Fe/PI(60)-1000-III-NH_3_, was successfully synthesized by the pyrolysis of an Fe-containing polyimide precursor prepared from PMDA and TAPB. This catalyst demonstrated good fuel cell performance and promising durability, especially with air as the cathode gas. The improvement of performance by employing small size NPM catalyst nanoparticles is due to the improvement of O_2_ diffusion in the catalyst layer. Further studies will be conducted to optimize the catalyst and increase the number of active sites, and to clarify the reaction mechanism.

## Methods

Polyimide nanoparticles with diameters of 100 nm were prepared by the precipitation polymerization of pyromellitic acid dianhydride (PMDA) and 4,4′-oxydianiline (ODA)^12^, which were purified by sublimation before use. A solution of PMDA in acetone was added to a solution of ODA and iron(III) acetylacetonate (Fe(acac)_3_, >98.0%, Dojindo) in acetone (>99.0%, Wako). The molar ratio of PMDA to ODA was 1:1 and the amount of the Fe species was 2 wt% with respect to the resulting polyimide. The mixture was stirred for 30 min at 0 °C. After evaporation of the solvent, the curing reaction proceeded by heating the poly(amic acid) at 200 °C under evacuation to obtain polyimide nanoparticles. Polyimide nanoparticles with diameters of 60 nm were prepared from PMDA and 1,3,5-tris(4-aminophenyl)benzene (TAPB, TCI, used as-received) in the presence of *N,N*-dimethyldodecylamine (TCI, used as-received) as a dispersant. A solution of TAPB in acetone (>99.0%, Wako) was added to a solution of PMDA, Fe(acac)_3_ and the dispersant (0.3 wt%) in acetone. The molar ratio of PMDA to TAPB was 1.5:1 and the amount of the Fe species was 2 wt% with respect to the resulting polyimide. The mixture was stirred for 30 min at 0 °C. After evaporation of the solvent, the curing reaction proceeded by heating the poly(amic acid) at 240 °C under evacuation to obtain polyimide nanoparticles.

The prepared polyimide nanoparticles were carbonized by multistep pyrolysis, as reported elsewhere^12^. Briefly, the Fe-containing polyimide precursor was heated at 600 °C for 5 h in a nitrogen atmosphere, and then heated again to 800 and 1000 °C for 1 h each in an ammonia atmosphere (50% balanced by nitrogen). The product was washed with conc. HCl after each of the heat treatments at 600 and 800 °C^13^.

The specific surface area and mesopore volume of the catalysts were determined by N_2_ adsorption using a volumetric adsorption measurement instrument (Belsorp-mini II, Bel Japan). The surface area and mesopore surface area were determined by the Brunauer-Emmett-Teller (BET) method and the Barrett-Joyner-Halenda (BJH) method, respectively. The total pore volume was calculated from the adsorption volume at *p*/*p*_0_ = 0.99. The C, H and N contents of the catalysts were determined using a CHN elemental analyzer (2400-II, Perkin Elmer). The Fe content was determined by wavelength dispersive spectrometry (WDS) using an electron probe micro analyzer (EPMA; JXA-8100, Jeol). A LiFH crystal was used for Fe detection. The EPMA measurement was conducted by analyzing a pellet prepared from the catalyst powder without any binder. X-ray photoelectron spectroscopy (XPS) was performed using a spectrometer (JPS-9010MC, Jeol) equipped with a monochromator and an Al anode at 12 kV and 25 mA. The binding energy was charge-corrected with respect to the C 1*s* signal at 284.6 eV derived from aromatic carbon. X-ray absorption near edge structure (XANES) was used for chemical state analysis of the Fe species. XANES measurements of the prepared catalysts and reference samples were conducted at the BL14B2 beamline (SPring-8, Japan). The incident X-ray was monochromatized by a Si(111) double crystal monochromator and the energy resolution (E/ΔE) of incident beam was ~10000. Fe K-edge XANES were recorded in the transmission mode with a gas-filled ion chamber at room temperature. Iron phthalocyanine (FePc), (FePc)_2_O^28^, FeO, α-Fe_2_O_3_, Fe_3_O_4_, and Fe foil were used as reference samples. The photon energy was calibrated with the absorption energy of the reference Fe foil (7117 eV). The XANES data were analyzed using the Athena and Artemis software^29^. The particle morphology was evaluated using field emission-scanning electron microscopy (S-5500, Hitachi).

The catalytic performance of the prepared catalyst was studied by fuel cell testing using a membrane electrode assembly (MEA) prepared with the Fe/PI(100)-1000-III-NH_3_ and Fe/PI(60)-1000-III-NH_3_ catalysts. The cathodes were fabricated by application of an ink consisting of the carbon-based catalyst and a Nafion binder (20 wt% DE2021CS, DuPont) onto a gas diffusion layer (GDL25BC, SGL) using an auto film applicator (Tester Sangyo). The anode was fabricated with a PtRu/Ketjen black catalyst (TEC61E54, Tanaka) in the same manner on a gas diffusion layer. The MEA was fabricated by pressing the electrodes and a Nafion membrane (NR211, DuPont) at 150 °C and 30 kg cm^−2^ for 2 min.

The MEA performance was tested at 80 °C by flowing fully humidified hydrogen (300 mL min^−1^) into the anode side and fully humidified oxygen or air into the cathode side (300 mL min^−1^). The absolute pressures of the anode and cathode compartments were maintained at 0.2 MPa, unless otherwise stated. *I*-*V* polarization curves were measured by recording the cell voltages after holding the current density with an electronic load unit (PLZ164WA, Kikusui) for 5 min at each value. Durability tests were conducted by holding the current density at 200 mA cm^−2^ and recording the cell voltage.

## Additional Information

**How to cite this article**: Nabae, Y. *et al.* Pt-free carbon-based fuel cell catalyst prepared from spherical polyimide for enhanced oxygen diffusion. *Sci. Rep.* **6**, 23276; doi: 10.1038/srep23276 (2016).

## Supplementary Material

Supplementary Information

## Figures and Tables

**Table 1:** Elemental composition and specific surface area of the polyimide derived catalysts.

Sample name	Elemental analysis (wt%)			EPMA (wt%)	Specific surface area (m^2^/g)		Total pore volume (cm^3^ g^−1^)
	C	H	N	Fe	*A*_BET_	*A*_meso_	
Fe/PI(100)-1000-III-NH_3_	84.0	1.2	2.6	1.1	1200	202	1.18
Fe/PI(60)-1000-III-NH_3_	91.3	trace	3.0	1.5	1217	233	1.34
