# Supplementary material for: Pt-free carbon-based fuel cell catalyst prepared from spherical polyimide for enhanced oxygen diffusion
Source: Sci Rep. 2016 Mar 18;6:23276. doi: 10.1038/srep23276 (PMC4796867; doi:10.1038/srep23276)
Supplement: Supplementary Information [file srep23276-s1.doc]

Supplementary Information

**Pt-free carbon-based fuel cell catalyst prepared from spherical polyimide for enhanced oxygen diffusion**

*Yuta Nabae, Shinsuke Nagata, Teruaki, Hayakawa, Hideharu Niwa, Yoshihisa Harada, Masaharu Oshima, Ayano Isoda, Atsushi Matsunaga, Kazuhisa Tanaka and Tsutomu Aoki*

Table S1. Relative composition ratio of Fe (%) by linear combination fitting analysis for Fe *K*-edge XANES spectra of the polyimide derived carbon catalyst. Error bars are within ±4.2%

|  | Fe foil | Fe_3_C | α-Fe_2_O_3_ | Fe_3_O_4_ | FeO | FePc | (FePc)_2_O |
| --- | --- | --- | --- | --- | --- | --- | --- |
| Fe/PI(100)-1000-III-NH_3_ | 24.1 | 20.9 | 23.6 | 1.3 | 5.4 | 18.6 | 6.1 |
| Fe/PI(60)-1000-III-NH_3_ | 27.6 | 15.8 | 27.0 | 4.8 | 0.8 | 18.7 | 5.3 |

Figure S1. N_2_ adsorption isotherms of the prepared samples.

Figure S2. XRD patterns of the prepared samples and reference JCPDS data.

Figure S3. Reaction scheme for oxygen reduction.
